# Supplementary material for: The interactome of intact mitochondria by cross-linking mass spectrometry provides evidence for coexisting respiratory supercomplexes
Source: Mol Cell Proteomics. 2017 Dec 8;17(2):216–32. doi: 10.1074/mcp.RA117.000470 (PMC5795388; doi:10.1074/mcp.RA117.000470)
Supplement: Supplemental Data [file supp_RA117.000470_133922_0_supp_23978_fzffwf.zip › spectra_annotation/mito_spectra_annotation/405-1-10-1-12-1.pdf]

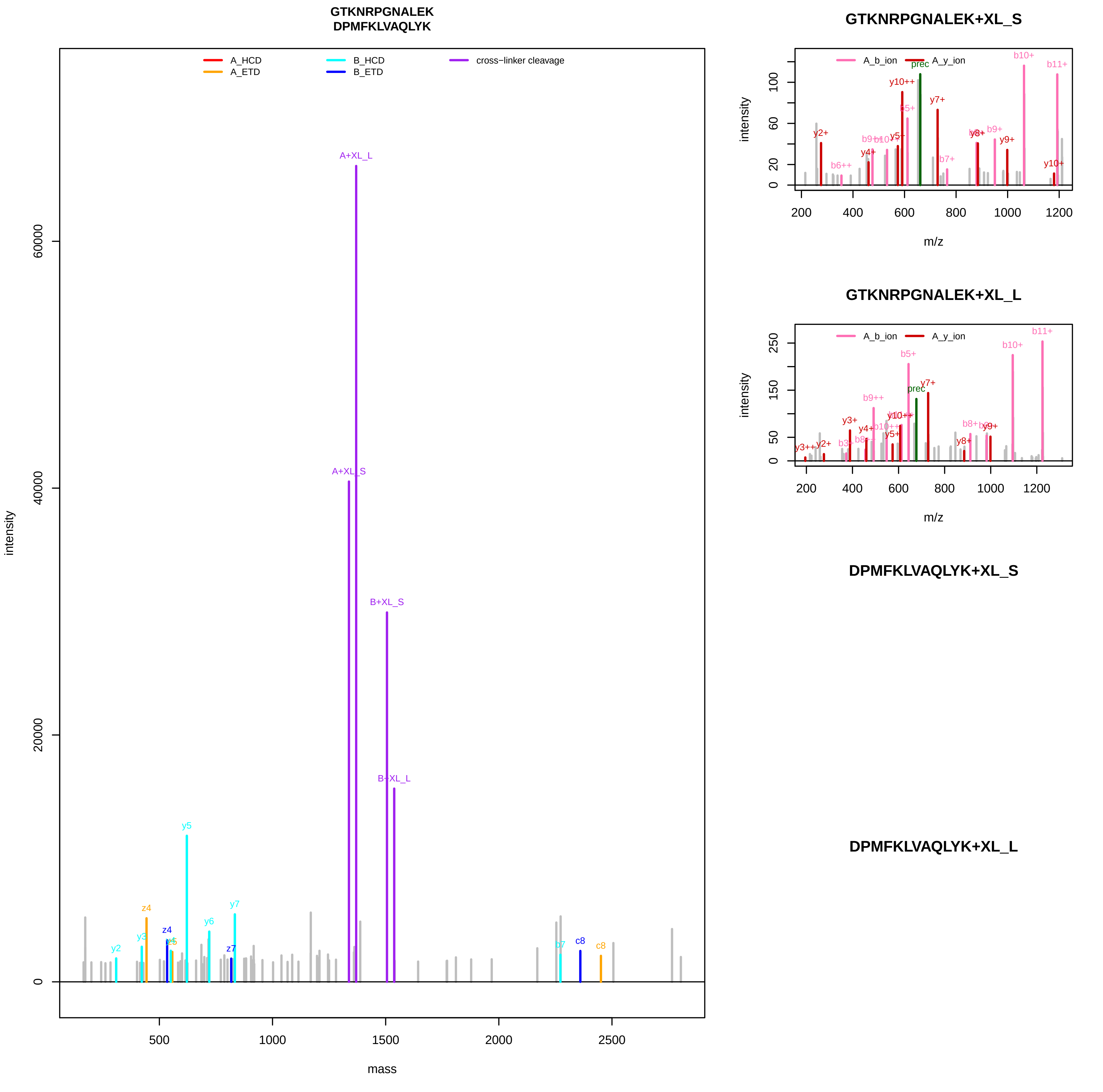
GTKNRPGNALEK
DPMFKLVAQLYK
A_HCD
A_ETD
B_HCD
B_ETD
cross−linker cleavage
A+XL_L
A+XL_S
B+XL_S
B+XL_L
y2
y3
z4
y5
y6
y7
z7
c8
intensity
mass
0
20000
40000
60000
500
1000
1500
2000
2500
GTKNRPGNALEK+XL_S
A_b_ion
A_y_ion
prec
y10++
y7+
y2+
b5+
b10+
b11+
b9+
y9+
y10+
b7+
b6++
y4+
y5+
y8+
m/z
GTKNRPGNALEK+XL_L
b5+
b11+
b10+
b9++
y3+
y4+
y7+
y9+
y8+
y2+
y3++
b8+
b3+
DPMFKLVAQLYK+XL_S
DPMFKLVAQLYK+XL_L
